# Dementia Care Training Regulations and Deficiencies of Care for Inappropriate Psychotropics Use in Nursing Homes

**DOI:** 10.1093/geroni/igaa057.524

**Published:** 2020-12-16

**Authors:** Jung Min Yoon, Alison Trinkoff, Carla Storr, Elizabeth Galik

**Affiliations:** 1 University of Maryland, Baltimore, Baltimore, Maryland, United States; 2 University of Maryland, Baltimore, Maryland, United States

## Abstract

Psychotropics are often used to manage behavioral and psychological symptoms of dementia (BPSD) in nursing homes (NHs), despite their adverse effects and lack of efficacy. NHs can be flagged for inappropriate psychotropics use as a deficiency of care (F-tag 758). To improve dementia care, 15 states require dementia-specific in-service training for NH nursing staff with specific training content and hours. The study aimed to relate the occurrence of F-758 citations to the presence of dementia-specific in-service training regulations, stratified by nurse staffing levels (<75th vs ≥75th percentile of nurse hours per resident day, HPRD). Certification and Survey Provider Enhanced Reporting data (n=14,548 NHs) from 2017-18 were used, containing 1,872 NHs with F-758 tags related to care of residents with dementia. NHs in states specifying training content and hours had significantly lower odds of receiving F-758 tags (OR=0.75, 95% CI=0.60-0.94). Among NHs with lower registered nurse HPRD, those in states regulating training content and hours had significantly lower odds of receiving F-758 tags (OR=0.66, 95% CI=0.49-0.89), with similar findings among NHs with lower certified nurse assistant HPRD (OR=0.69, 95% CI=0.51 52-0.91 92). This study found that required dementia-specific in-service training may be helpful in facilities with lower staffing. It is recommended that states develop more comprehensive, robust dementia care training regulations for NH nursing staff.

